# Current knowledge of scoliosis in physical therapy students trained in the United States

**DOI:** 10.1186/1748-7161-9-S1-O64

**Published:** 2014-12-04

**Authors:** Shawn Drake, Michael Glidewell, Jerrica Thomas

**Affiliations:** 1 Arkansas State University, Jonesboro, AR, USA

## Background

Previous research suggests that the average knowledge of idiopathic scoliosis among physical therapy students in Poland may be unsatisfactory with respect to SOSORT guidelines, which provide the most up-to-date conservative treatment options. A similar study is warranted for students enrolled in physical therapy schools in the United States.

## Aim

The purpose of this study is to determine basic knowledge of idiopathic scoliosis in physical therapy students trained in the United States.

## Design

Exploratory research / Questionnaire.

## Methods

A 10-question survey, which included knowledge of 2011 SOSORT Guidelines, was developed for this study. The sample selected for this study included 130 randomly selected physical therapy schools offering the Doctor of Physical Therapy degree in the United States. Physical therapy program directors receiving the research study invitation were asked to forward the anonymous online survey link (Qualtrics Survey Research Suite, Provo, UT) to students meeting the inclusion criteria. The details of the number of students who actually received the study invitation are unknown. The survey link closed after 4-weeks of data collection.

## Results

One hundred ninety-two students (192) initiated the survey. A total of 182 students completed consent and met inclusion criteria for the study. Four respondents did not complete all answers to the questionnaire and were not included in final analysis. Therefore, data reflects responses from 178 physical therapy students across the United States. Only 15 students (8%) answered 70% of the survey questions correctly.

## Conclusions

Results from this study indicate that physical therapy students within the United States are not trained in knowledge related to the 2011 SOSORT Guidelines.

